# Comparison of Short- and Long-Term Prognosis between ST-Elevation and Non-ST-Elevation Myocardial Infarction

**DOI:** 10.3390/jcm10020180

**Published:** 2021-01-07

**Authors:** Frédéric Bouisset, Jean-Bernard Ruidavets, Jean Dallongeville, Marie Moitry, Michele Montaye, Katia Biasch, Jean Ferrières

**Affiliations:** 1 Department of Cardiology, Rangueil University Hospital, 31059 Toulouse, France; jean.ferrieres@univ-tlse3.fr; 2 Department of Epidemiology, INSERM UMR 1027, 31000 Toulouse, France; jeanbernard.ruidavets@gmail.com; 3 Institut Pasteur de Lille, Department of Epidemiology and Public Health, Inserm-U1167, 59000 Lille, France; jean.dallongeville@pasteur-lille.fr (J.D.); michele.montaye@pasteur-lille.fr (M.M.); 4 Faculty of Medicine, Department of Epidemiology and Public Health, University of Strasbourg, 67081 Strasbourg, France; marie.moitry@chru-strasbourg.fr (M.M.); katia.Biasch@unistra.fr (K.B.); 5 Department of Public Health, Strasbourg University Hospital, 67085 Strasbourg, France

**Keywords:** acute coronary syndrome, coronary heart disease, prognosis

## Abstract

Background: Available data comparing long-term prognosis according to the type of acute coronary syndrome (ACS) are scarce, contradictory, and outdated. Our aim was to compare short- and long-term mortality in ST-elevated (STEMI) and non-ST-elevated myocardial infarction (non-STEMI) ACS patients. Methods: Patients presenting with an inaugural ACS during the year 2006 and living in one of the three areas in France covered by the Monitoring of Trends and Determinants in Cardiovascular Disease (MONICA) registry were included. Results: A total of 1822 patients with a first ACS—1121 (61.5%) STEMI and 701 (38.5%) non-STEMI—were included in the study. At the 28-day follow-up, the mortality rates were 6.7% and 4.7% (*p* = 0.09) for STEMI and non-STEMI patients, respectively, and after adjustment of potential confounding factors, the 28-day probability of death was significantly lower for non-STEMI ACS patients (Odds Ratio = 0.58 (0.36–0.94), *p* = 0.03). At the 10-year follow-up, the death rates were 19.6% and 22.8% (*p* = 0.11) for STEMI and non-STEMI patients, respectively, and after adjustment of potential confounding factors, the 10-year probability of death did not significantly differ between non-STEMI and STEMI events (OR = 1.07 (0.83–1.38), *p* = 0.59). Over the first year, the mortality rate was 7.2%; it then decreased and stabilized at 1.7% per year between the 2nd and 10th year following ACS. Conclusion: STEMI patients have a worse vital prognosis than non-STEMI patients within 28 days following ACS. However, at the 10-year follow-up, STEMI and non-STEMI patients have a similar vital prognosis. From the 2nd year onwards following the occurrence of a first ACS, the patients become stable coronary artery disease patients with an annual mortality rate in the 2% range, regardless of the type of ACS they initially present with.

## 1. Introduction

Acute coronary syndrome (ACS) patients are categorized into two distinct presentations according to the initial electrocardiogram (ECG): ST-elevation myocardial infarction (STEMI) and non-STEMI ACS [1]. Both types of ACS share the same pathophysiology: in most cases, a thrombus is formed in a coronary artery after the rupture or erosion of a vulnerable atherosclerotic plaque [2]. A totally occluding thrombus typically leads to STEMI [1], whereas a partial occlusion, or occlusion in the presence of collateral circulation, results in non-STEMI [3].

Among previous studies that compared vital prognosis according to the type of ACS, many were limited by a relatively short follow-up or lack of adjustment [4,5,6,7]. Some others did have long-term follow-up but reported conflicting results. These studies included patients in the 1990s or the early 2000s, and management of ACS has dramatically improved since then, potentially affecting long-term prognosis [8,9,10,11].

The data presented here were collected from a population-based registry—the Monitoring of Trends and Determinants in Cardiovascular Disease (MONICA) registry—which prospectively include every incidental case of ACS reported in three areas of France, with the aim to compare presentation, management, and short- and long-term prognosis according to the type of initial ACS (STEMI and non-STEMI).

## 2. Methods

### 2.1. Population and Inclusion Criteria

During the year 2006, all patients aged 35–74 years hospitalized for a first ACS in one of the three geographic areas covered by the three French registries of myocardial infarction (Northern, Northeastern, and Southwestern France) were prospectively included. The standardized registration methods used in each of these registries derived from the Monitoring of Trends and Determinants in Cardiovascular Disease (MONICA) project, a study focused on the management of acute coronary events [12,13]. Only patients presenting with no previous history of myocardial infarction or angina pectoris were considered. A previous history of coronary events was defined on the basis of anamnestic or documented information from the patients’ medical records.

### 2.2. Data Collection

Initial clinical presentation, electrocardiogram (ECG) features, cardiac enzymes (CPK, CK-MB, or troponin levels), left ventricular ejection fraction (LVEF), and invasive management (coronary angiography ± myocardial revascularization) were systematically transcribed from medical records. An exhaustive prospective record of treatments before the onset of the event, during hospitalization, and at discharge was completed for each patient. Patients were then categorized into two groups: ST-elevation myocardial infarction (STEMI) and non-ST-elevation myocardial infarction (non-STEMI). 

ACS was classified as STEMI ACS if presenting, on the qualifying 12-lead ECG, either an ST segment elevation ≥1 mm on at least two contiguous peripheral leads, an ST segment elevation ≥2 mm on at least two contiguous precordial leads, a presumed new Q wave, or a left bundle branch block. Acute coronary syndrome was defined as non-STEMI ACS in the absence of ST segment elevation criteria with the presence of significant cardiac enzyme elevation.

### 2.3. Patients’ Follow-up

Vital status was collected at 28 days, and patients were followed up for ten years until 31 December 2015, using the Répertoire National d’Identification des Personnes Physiques (National Identification Register of Private Individuals—RNIPP) register, a French national database that records, on a yearly basis, the deaths of all French citizens living inside or outside the French territory. This method allowed for obtaining the vital status of 100% of the patients included in this study.

### 2.4. Statistical Analysis

We first described and compared the main characteristics of the patients according to ACS classification. Categorical variables were compared between groups using the χ^2^ test (or Fisher’s exact test when necessary). Student’s *t*-tests were used to compare the distribution of continuous data (Mann–Whitney tests were used when the distribution of the continuous variable departed from normality or when homoscedasticity was rejected). Follow-up was scheduled at 28 days and 10 years. Cumulative survival curves were drawn using the Kaplan–Meier method and were compared using the log-rank test for all-cause mortality. The associations between baseline variables and death were assessed using logistic regression to adjust for predictors of short-term mortality and Cox proportional hazard regression analysis to adjust for predictors of long-term mortality. We assessed the proportionality assumption using cumulative sums of martingale-based residuals. We performed regression analyses with polynomial models (quadratic and cubic) to examine for possible nonlinear relations between continuous variables and mortality. Two long-term analyses were performed over 10 years, one including all patients and the other excluding patients deceased at 28 days. All statistical analyses were carried out using the statistical SAS software package (SAS 9.4 Institute, Cary, NC, USA) and STATA 14.2 (StataCorp, College Station, TX, USA). All tests were two-tailed, and statistical significance was assumed at *p*-value < 0.05.

## 3. Results

A total of 1822 patients aged 35–74 presenting with a first acute coronary syndrome and no history of coronary event were included over the year 2006 in the three French areas. Among them, 1121 presented with a STEMI (61.5%) and 701 with a non-STEMI (38.5%). The proportion of male patients was 78.2% in the STEMI group, significantly higher than in the non-STEMI group (73.2%, *p* = 0.02), and the mean age was 56.6 ± 10.4 and 60.3 ± 9.9 years, respectively. Three hundred and eighty patients (20.8%) died during the ten-year follow-up. One hundred and eight patients (5.9%) died during the first 28 days (6.7% in the STEMI group vs. 4.7% in the non-STEMI, *p* = 0.09), and 272 (15%) died during the following ten years (13.9% STEMI and 19.0% non-STEMI, *p* = 0.005) (Table 1).

Table 1 provides a description of the population according to the type of ACS. The left part of the table presents a comparison of clinical presentation of STEMI and non-STEMI patients considering the entire cohort. The right part presents the same comparison after the exclusion of the patients who died during the first 28 days after the index event.

The use of cardiovascular drugs and revascularization procedures during the acute phase management was significantly different among STEMI and non-STEMI patients (Table 2). In the STEMI group, the prescription of ACE inhibitors, antiarrhythmic drugs, and percutaneous coronary interventions was significantly higher, whereas calcium blockers, angiotensin II blockers, and coronary artery bypass surgery were less prescribed. Regarding treatment at discharge, significantly higher proportions of STEMI patients were treated with antiplatelet agents, beta blockers, and statin drugs, while a small number of them received calcium blockers and angiotensin II blockers. The prescription of cardiac rehabilitation was two times higher in the STEMI group (44.7% versus 22.6% in the non-STEMI group, *p* = 0.001).

Table 2 provides a description of the therapeutic management and anatomical status according to the type of ACS. The left part of the table presents a comparison of therapeutic management during the pre- and in-hospitalization period and the anatomical status of STEMI and non-STEMI patients, considering the entire cohort. The right part compares the medical therapy prescribed at discharge and strategies of revascularization and cardiac rehabilitation of STEMI and non-STEMI patients and after exclusion of patients deceased during the first 28 days after the index ACS.

A comparison between the ACS patients’ characteristics and the number of deaths in each group according to the follow-up duration is described in Table 3. In the short term, the patients living in Northern and in Northeastern France were at a higher risk of death than those living in the southwestern areas. Age, signs of severity at presentation (cardiac arrest, cardiogenic shock, Killip ≥ 2, syncope), past medical history, and cardiovascular risk factors were positively and significantly associated with mortality at 28 days. LVEF was negatively associated with mortality. At long-term follow-up, after the patients deceased within the first 28 days were excluded, age and clinical signs of severity at onset remained positively associated with mortality. Preserved LVEF, recommended treatment for ACS (in accordance with European guidelines) at discharge, and prescription of cardiac rehabilitation were negatively associated with long-term mortality.

Table 3 presents factors associated with the occurrence of death during follow-up. The left part of the table focuses on the occurrence of death within 28 days after the index ACS. The right part focuses on the occurrence of death within 10 years, excluding or not the patients deceased within 28 days after the index ACS.

**Figure 1:**
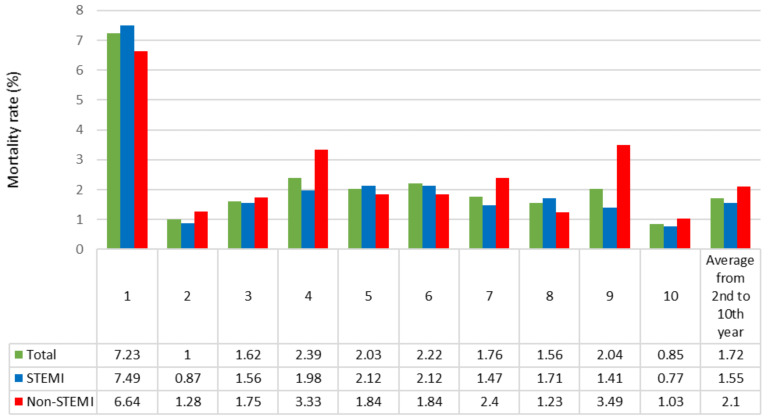
Annual mortality rates during follow-up according to the type of acute coronary syndrome.

The high mortality rate (7.2%) in the first year mostly resulted from an early mortality within 28 days following the index cardiac event (Figure 1). Thereafter, the annual mortality rates of ACS decreased and remained fairly stable, varying between 1% and 2%, with higher annual rates in non-STEMI than in STEMI patients.

Figure 1 demonstrates that after the first year, characterized by a mortality rate in the range of 7%, the mortality rate of the ACS population reached the annual rate in the range of 2%, regardless of the type of ACS initially presented.

**Figure 2:**
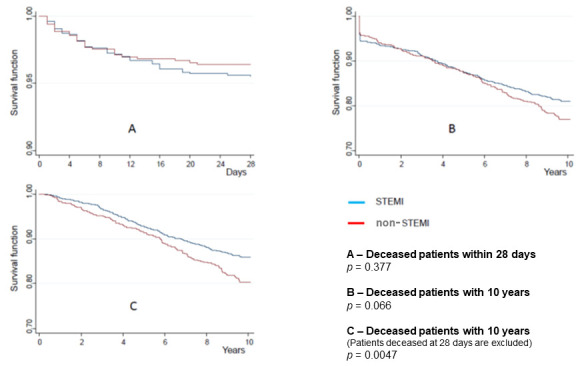
Cumulative survival according to the type of acute coronary syndrome.

As shown in Figure 2, the mortality rate at 28 days was higher in the STEMI group, particularly after the fourteenth day, while it became significantly higher over the 10 following years in the non-STEMI group, after patients deceased within 28 days were excluded from the data analysis.

Panel A of Figure 2 shows that STEMI patients presented a higher crude mortality at the 28-day follow-up after the index ACS. Panels B and C show that non-STEMI patients presented a higher crude mortality at the 10-year follow-up after the index ACS.

Table 4 shows comparisons between STEMI and non-STEMI ACS and patients’ mortality according to the follow-up duration. Within the first 28 days, the risk of death in non-STEMI patients was lower compared with that of those presenting with STEMI, with an Odds Ratio of 0.69 (95% CI (0.45–1.05)) in univariate analysis. After adjustment for age, gender, and geographical area, the OR was 0.58 (0.38–0.89, *p* = 0.02) and remained significant after further adjustment for clinical signs of severity, cardiovascular drugs prior to hospitalization, and LVEF with a Hazard Ratio of 0.58 (95% CI (0.36–0.94), *p* = 0.03). The risk of death in non-STEMI patients who survived the first 28 days was significantly higher than in STEMI patients, with an HR of 1.41 (95% CI (1.11–1.49), *p* = 0.005). After adjustment for age, gender, and medical center, the difference in the risk of death became nonsignificant (HR = 1.12, 95% CI (0.88–1.42), *p* = 0.43) and remained as such after further adjustments for clinical signs of severity, cardiovascular drugs before hospitalization, left ventricular ejection fraction, cardiovascular treatments at discharge, and cardiac rehabilitation (HR = 1.07, 95% CI (0.83–1.38), *p* = 0.59).

## 4. Discussion

The present study compares the clinical characteristics, short- and long-term vital prognosis, and medical management of STEMI vs. non-STEMI ACS in a cohort of patients living in the three areas of France covered by the MONICA registry. At the 28-day follow-up, the vital prognosis was worse for STEMI patients, but similar for both groups at the 10-year follow-up. After the first year following the index ACS, the mortality rate was in the 7% range, while it then decreased and stabilized to an annual rate within a 2% range—similar to the range usually observed in stable coronary artery disease patients.

### 4.1. Clinical Characteristics and Management

Our cohort found the usual differences observed between STEMI and non-STEMI populations: non-STEMI patients were older, more often female, and had more severe coronary lesions. These two populations also differed in their medical management. In the acute phase, non-STEMI patients were less invasively explored (89.3% vs. 94.8%, *p* < 0.001) and treated using reperfusion therapy (58.5% vs. 76.6%, *p* < 0.001) for Percutaneous Coronary Intervention. They also received statin therapy (85.1% vs. 81.2) and ACE inhibitors (73.8 vs. 61.2%) less frequently. After the acute stage, non-STEMI patients were half as likely to benefit from cardiac rehabilitation as STEMI patients. Since these differences influence the prognosis, it appeared critical to adjust the comparison between both groups on these parameters to accurately assess the differences of prognosis.

### 4.2. Short-Term Follow-up (28 Days)

In the present study, crude mortality rates at 28 days were 6.7% and 4.7% for STEMI and non-STEMI patients, respectively. These results are concordant with those observed in France for the same period in a nationwide registry [14]. After complete adjustment, short-term mortality remained significantly lower for non-STEMI patients (0.58, 95% CI (0.36–0.94), *p* = 0.03). This higher, adjusted short-term mortality risk in STEMI patients has been previously reported in several cohorts [11,15,16] and can be attributed to a higher incidence of mechanical complications in the acute stage. Cardiogenic shock [17] and mechanical complications [18], although rare, are more frequently described in STEMI than in non-STEMI patients, and associated with a very high mortality rate [17,18].

### 4.3. Long-Term Follow-up

At the one-year follow-up, the mortality rates were 7.5% and 6.6% for STEMI and non-STEMI, respectively. After the first year, the annual mortality rates were 1.6% and 2.1% for STEMI and non-STEMI patients, respectively. These annual mortality rates are concordant with those observed in the Netherlands [19] and Sweden [20] in cohorts of patients followed after an ACS treated using PCI and reach the expected mortality rate in stable coronary artery disease patients [21]. This supports the theory that patients who present with an acute coronary syndrome can be considered stable, integrating the population of chronic coronary syndrome patients, one year following the index event.

At the ten-year follow-up, the comparison of the mortality rate according to the type of ACS showed, after complete adjustment, a similar risk of death between both populations (Table 4). Previous studies had contradictory results, some suggesting that non-STEMI patients have a worse long-term prognosis [11], while others reported that STEMI patients have a worse prognosis [9]. Our results, showing no differences in the long-term vital prognosis among both groups, are coherent, since both diagnoses (STEMI and non-STEMI) reflect the same pathological process. Adjustment appears to be critical in the data analysis presented here, as non-STEMI patients were older than STEMI patients, potentially explaining a large part of the differences observed in non-adjusted results. The initial presentation was included in our adjustment, as it had been demonstrated to be an independent predictor of long-term mortality in both non-STEMI and STEMI populations [22].

Our results, showing, after adjustment, a similar long-term risk of death in both types of ACS, reflect that they are two different faces of the same pathology. The underlying risk factors and pathophysiological mechanisms are common in both types of ACS [23]. It therefore seems consistent to observe the same long-term vital prognosis for both types of ACS. The borderline between these two forms of ACS is blurred. European guidelines on non-STEMI recommend considering for emergent coronary angiography non-STEMI patients presenting with ongoing pain [24]. This recommendation reflects that a proportion of patients present with a totally occluded epicardial coronary, jeopardizing a large amount of myocardial muscle, especially in the case of circumflex artery occlusion, in the absence of ST elevation seen in the ECG. Therefore, an ECG does not always predict the completeness of coronary occlusion. The definition of STEMI on the ECG is an evolving concept: the latest ESC guidelines on STEMI included de novo right bundle branch block as a new criterion of STEMI [1].

Although our study included relatively young patients (under 75 years), the mortality rate at the 10-year follow-up remained relatively high, at 20.8%. This underlines the fact that, despite critical improvements in the management of acute coronary syndromes in the recent years—both in the acute stage and after—this pathology remains severe and fatal.

### 4.4. Limitations

The MONICA registry does not record information on renal function and cardiovascular risk factors at baseline, and registration is limited to patients aged 35–74. However, and unlike most previous studies that compared STEMI and non-STEMI prognosis, this registry includes all patients, regardless of the hospitalization department and whether they have myocardial revascularization (by Coronary Artery Bypass Grafting or Percutaneous Coronary Intervention) or remain only medically treated. The present analysis concerned only all-cause mortality, considering that we did not have data on the causes of death. It is likely that a small proportion of patients presenting with a myocardial infarction with non-obstructive coronary arteries (MINOCA) were included in this registry. However, since this pathophysiological concept was not clearly established at the time of recruitment (2006), these patients have not been clearly identified.

## 5. Conclusions

In this contemporary, population-based registry, we observed that vital prognosis is worse in the short term in STEMI than in non-STEMI patients, but that it becomes similar among both groups in the long term, after complete adjustment for potential confounding factors. After the first year following the index acute coronary syndrome, the annual mortality rate of the patients reached 1.7%, which is the same rate as in the population of stable coronary disease patients.

## Figures and Tables

**Table 1:** Patients’ characteristics according to the type of acute coronary syndrome.

	Entire Cohort			After Exclusion of Patients Deceased within 28 Days		
	STEMI (*n* = 1121)	Non-STEMI (*n* = 701)	*p*-Value	STEMI (*n* = 1046)	Non-STEMI (*n* = 668)	*p*-Value
Age (years)	56.6 ± 10.4	60.3 ± 9.9	0.001	56.2 ± 10.3	60.1 ± 9.9	0.001
Male gender	78.2	73.2	0.02	78.5	72.9	0.008
Geographical area			0.31			0.47
Northern France	29.0	31.1		34.3	35.8	
Northeastern France	35.9	32.4		35.4	32.5	
Southwestern France	35.2	36.5		30.1	31.7	
Symptom presentation						
Cardiac arrest	7.5	1.2	0.001	5.1	0.8	0.001
Cardiogenic shock	4.9	3.1	0.07	2.6	1.4	0.09
Syncope	4.0	3.7	0.75	3.9	2.8	0.24
Killip ≥2	12.0	16.3	0.01	10.7	15.3	0.006
At least 1 clinical sign of severity at presentation	24.6	23.0	0.43	20.5	19.5	0.62
LVEF			0.001			0.001
≥50%	68.7	81.3		70.8	82.2	
35–50%	23.5	10.7		23.3	10.8	
<35%	4.8	4.6		3.9	3.9	
Unmeasured	3.0	3.4		2.0	3.1	
Time from symptoms to hospitalization <4 h (%)	55.8	43.8	0.001	-	-	
All-cause deaths						
Within 28 days	6.7	4.7	0.09			
Within 10 years	19.6	22.8	0.11	13.9	19.0	0.005

LVEF, left ventricular ejection fraction.

**Table 2:** Therapeutic management and anatomical status according to the type of acute coronary syndrome.

	Entire Cohort			After Exclusion of Patients Deceased within 28 Days		
	Pre- and In-Hospitalization			At Discharge		
	STEMI (*n* = 1121)	Non-STEMI (*n* = 701)	*p*-Value	STEMI (*n* = 1046)	Non-STEMI (*n* = 668)	*p*-Value
Medical therapy						
Parenteral anticoagulation	94.3	93.9	0.71	10.5	13.9	0.04
Antiplatelet therapy	96.8	96.4	0.69	96.3	93.0	0.003
Nitrates	61.0	66.8	0.02	10.3	12.4	0.18
Beta blockers	85.3	82.5	0.11	86.1	76.8	0.001
Calcium blockers	17.1	28.7	0.001	8.3	17.2	0.001
ACE inhibitors	73.8	61.2	0.001	75.6	63.0	0.001
ARBs	6.9	10.8	0.003	5.6	10.5	0.001
Inotropic agents	12.5	10.1	0.13	1.0	0.8	0.66
Diuretic agents	29.2	29.0	0.93	16.5	19.9	0.07
Other antihypertensive therapy	2.5	5.8	0.002	1.0	3.0	0.002
Statin therapy	85.1	81.2	0.03	90.4	85.0	0.001
Antidiabetic therapy	20.4	24.5	0.04	13.5	19.2	0.002
Antiarrhythmic therapy	15.1	10.7	0.008	2.9	3.4	0.51
Invasive management						
Coronary angiography	94.8	89.3	0.001	5.8	5.9	0.99
Pacemaker	2.4	1.3	0.10	0.4	0.9	0.21 *
Number of diseased vessels			0.001			
One vessel	46.9	37.5		-	-	-
Two vessels	26.5	20.0		-	-	-
Three vessels	16.1	22.2		-	-	-
Unknown	5.4	9.6		-	-	-
Unassessed	5.2	10.7		-	-	-
Reperfusion therapy						
Thrombolysis	7.0	0.3	0.001	-	-	-
PCI	76.6	58.5	0.001	6.2	5.8	0.76
Coronary artery bypass surgery	2.7	6.3	0.001	2.5	2.7	0.79
Cardiac rehabilitation	-	-	-	41.7	22.6	0.001

* Fisher’s exact test. ACE, angiotensin-converting enzyme; ARB, angiotensin II receptor blocker; PCI, percutaneous coronary intervention.

**Table 3:** Predictors of death according to the follow-up duration.

	Patients Deceased within 28 Days (*n* = 108)			Patients Deceased within 10 Years (*n* = 380) *			Patients Deceased within 10 Years (*n* = 272) **		
	HR	95% CI	*p*-Value	HR	95% CI	*p*-Value	HR	95% CI	*p*-Value
Age (years)	1.05	1.03–1.07	0.001	1.06	1.05–1.08	0.001	1.07	1.06–1.09	0.001
Northern France vs. Southwestern France	3.28	1.80–5.99	0.001	1.46	1.14–1.89	0.004	1.17	0.88–1.56	0.30
Northeastern France vs. Southwestern France	2.71	1.46–5.01	0.002	1.16	0.89–1.52	0.27	0.91	0.67–1.24	0.56
Women vs. men	1.02	0.65–1.61	0.93	1.26	1.01–1.57	0.05	1.35	1.04–1.76	0.03
Clinical signs of severity ^a^	24.7	14.1–43.1	0.001	3.67	3.00–4.49	0.001	2.04	1.58–2.63	0.001
Cardiovascular drugs prior to hospitalization	2.08	1.37–3.16	0.001	2.33	1.87–2.90	0.001	2.46	1.89–3.19	0.001
LVEF 35–50% vs. ≥50%	1.76	1.05–2.94	0.04	1.60	1.25–2.05	0.001	1.57	1.18–2.08	0.002
LVEF <35% vs. ≥50%	7.17	4.01–12.8	0.001	3.86	2.79–5.35	0.001	3.05	2.00–4.67	0.001
Recommended treatment at discharge ^b^							0.80	0.69–0.93	0.004
Cardiac rehabilitation							0.53	0.40–0.70	0.001

* Entire cohort; ** exclusion of patients deceased within 28 days; ^a^ cardiac arrest, cardiogenic shock, Killip ≥ 2, syncope; ^b^ antiplatelet therapy plus statin therapy plus beta blockers; LVEF: left ventricular ejection fraction, HR: Hazard Ratio

**Table 4:** Unadjusted and adjusted comparison of survival according to the type of acute coronary syndrome and follow-up duration.

	Patients Deceased within 28 Days (*n* = 108), 5.93%			Patients Deceased within 10 Years (*n* = 380), 20.86% *			Patients Deceased within 10 Years (*n* = 272), 15.87% **		
	OR	95% CI	*p*	HR	95% CI	*p*	HR	95% CI	*p*
Non-STEMI vs. STEMI ^a^	0.69	0.45–1.05	0.09	1.16	0.95–1.43	0.15	1.41	1.11–1.79	0.005
Non-STEMI vs. STEMI ^b^	0.58	0.38–0.89	0.02	0.94	0.77–1.16	0.56	1.12	0.88–1.42	0.43
Non-STEMI vs. STEMI ^c^	0.57	0.36–0.89	0.02	0.95	0.77–1.18	0.66	1.15	0.90–1.47	0.27
Non-STEMI vs. STEMI ^d^	0.58	0.36–0.94	0.03	0.96	0.77–1.18	0.67			
Non-STEMI vs. STEMI ^e^							1.07	0.83–1.38	0.59

* Total patients deceased within 10 years; ** exclusion of patients deceased within 28 days; ^a^ unadjusted; ^b^ adjusted for age, gender, and region; ^c^ further adjustment for LVEF; ^d^ further adjustment for clinical signs of severity, treatment before hospitalization; ^e^ further adjustment for drugs at discharge, rehabilitation. OR: Odds Ratio

## Data Availability

The data presented in this study are available on request to Santé Publique France.
